# Levothyroxine sodium tablets reversed Hashimoto thyroiditis-induced kidney injury, muscle injury, and lipid metabolism disorder: A case report and literature review

**DOI:** 10.1097/MD.0000000000039190

**Published:** 2024-08-09

**Authors:** Xuesong Li, Chenxiang Cao

**Affiliations:** a School of Clinical Medicine, Tsinghua University, Beijing, China; b Department of Endocrine and Metabolism, Beijing Tsinghua Changgung Hospital, Beijing, China.

**Keywords:** Hashimoto thyroiditis, kidney injury, levothyroxine sodium tablets, lipid metabolism disorder, muscle injury

## Abstract

**Rationale:**

Hashimoto thyroiditis (HT), a common cause of hypothyroidism, has shown an increasing incidence in recent years, particularly among women. In addition to the common complications such as lipid metabolism disorders, patients with HT may also experience some serious complications, acute kidney injury and severe muscle damage for instance. This article explored the effectiveness of levothyroxine sodium tablets (L-T4) replacement therapy in severe complications of hypothyroidism, including treatment dosage, duration of complication recovery, and whether additional treatment is needed.

**Patient concerns, Diagnoses, and Interventions:**

We described a case of a 52-year-old woman with HT who exhibited kidney injury, muscle injury, and lipid metabolism disorders. The increased levels of serum creatinine, creatine kinase, cholesterol, triglyceride, low density lipoprotein cholesterol, high density lipoprotein cholesterol, and the decreased levels of estimated glomerular filtration rate were obviously observed. This patient was started on L-T4 (75 and 100 µg, alternate).

**Outcomes and Lessons:**

Following a two-month treatment, the serum creatine kinase level decreased to within normal range. The estimated glomerular filtration rate level was restored, and the serum creatinine level was down-regulated, although slightly higher than the normal range. L-T4 partially reversed HT-induced the disorders of muscle, renal function, and lipid profile of this patient and remarkably alleviated her HT-related symptoms.

## 1. Introduction

Hashimoto thyroiditis (HT), also known as autoimmune thyroiditis, is a chronic inflammatory of thyroid firstly described by Hashimoto in 1912.^[1]^ The exact incidence of HT in practice is unclear but is similar to that of Graves’ disease with a growing trend.^[2]^ The etiopathogenesis of HT is still incompletely comprehended. Classically, patients with HT always occurs as a painless, hard, and diffuse enlargement of the thyroid gland accompanying hypothyroidism. HT is well characterized by the production of autoantibodies like the most common antithyroid peroxidase antibodies and anti-thyroglobulin antibodies and with lymphocyte infiltration.^[3]^ The symptoms of HT patients are various but not specific, including fatigue, more sensitivity to coldness, weakness and increase in body weight, which may be due to extensive involvement of more systems.^[4]^ Patients with HT show distinct manifestations and pathophysiologic features depending on the severity of HT.

Herein, we reported a typical case of a 52-year-old woman with a diagnosis of HT with kidney injury, muscle injury, and lipid metabolism disorders who was treated with levothyroxine sodium tablets (L-T4).

## 2. Case report

A 52-year-old woman presented to our clinic with complaints of weakness, fatigue, afraid of cold, and myodynia on March 19, 2023. She was diagnosed with primary hypothyroidism 2 weeks prior at another hospital and received oral replacement therapy with 50 µg/day of L-T4. She had no history of chronic diseases such as nephropathy, hepatitis, muscular disease, hypertension, coronary heart disease, diabetes, or history of drug allergy. She also denied having a history of infectious diseases and blood transfusions. Family history of any thyroid diseases was unremarkable. Physical examination revealed that the thyroid gland is diffusely enlarged, tough, and hard in texture, without adhesion to surrounding tissues, and can move up and down with swallowing. The remainder of her physical examination was unremarkable.

We performed comprehensive accessory examinations for this patient. The thyroid function showed that serum levels of total serum triiodothyronine was 0.30 nmol/L (1.3–3.1 nmol/L), free serum triiodothyronine (FT3) was 0.96 pmol/L (3.1–6.8 pmol/L), total serum tetraiodothyronine was 18.60 nmol/L (66–181 nmol/L), free serum tetraiodothyronine (FT4) was 1.72 pmol/L (12–22 pmol/L), and thyroid stimulating hormone (TSH) was 176.00 μIU/mL (0.27–4.20 μIU/mL). Both antithyroid peroxidase antibodies and anti-thyroglobulin antibodies were seropositive at >600.00 KIU/L (<34 KIU/L) and >4300.00 IU/mL (0–115 KIU/L), respectively. Meanwhile, the serum level of creatine kinase (CK) was 952 U/L (40–200 U/L). Renal function test showed serum creatinine (Scr) was 89.00 μmol/L (41–73 μmol/L), and estimated glomerular filtration rate (eGFR) was 64.401 mL/min/1.73 m^2^. Lipid profile exhibited cholesterol (CHOL) was 10.95 mmol/L (<5.2 mmol/L), triglyceride (TRIG) was 1.93 mmol/L (<1.7 mmol/L), high density lipoprotein cholesterol (HDL-c) was 1.55 mmol/L (>1.0 mmol/L), and low density lipoprotein cholesterol (LDL-c) was 9.06 mmol/L (<3.4 mmol/L). Thyroid ultrasonography was performed and unraveled diffusely decreased echogenicity. Later, the dosage of L-T4 was increased up to 75 μg qd. On April 20, 2023, her serum levels of FT4 and FT3 were 16.3 pmol/L and 3.89 pmol/L, respectively, and serum TSH was 21.7 μIU/mL. The serum level of CK was decreased to normality. The level of eGFR was recovered and the serum level of Cre was down-regulated though slighter higher than that of normal. Lipid profile showed improved test results with CHOL of 10.95 mmol/L, TRIG of 1.93 mmol/L, and LDL-c of 9.06 mmol/L. She was prescribed to start on L-T4 (75 and 100 µg, alternate). On May 17, 2023, the thyroid function, renal function, and lipid metabolism of this patient exhibited a continuous trend in improvement with L-T4 replacement therapy. At a total 2-month follow-up in outpatient setting, she improved dramatically with a remarkable alleviation of her signs and symptoms. Collectively, L-T4 reversed HT-induced kidney injury, muscle injury, and lipid metabolism disorder to some content (Table 1).

**Table 1:** Changes in the levels of thyroid function, renal function, lipid profile, and CK during the whole L-T4 replacement therapy.

	March 20, 2023	April 19, 2023	May 17, 2023
Cre (41–73 µmol/L)	89.0	77.0	76.0
eGFR (ml/min/1.73 m^2^)	64	76	77
CHOL (<5.2 mmol/L)	11.0	6.2	5.3
TRIG (<1.7 mmol/L)	1.9	1.4	1.3
LDL-c (<3.4 mmol/L)	9.1	4.6	3.9
HDL-c (>1.0 mmol/L)	1.6	1.3	1.1
CK (40–200 U/L)	952.0	117.0	43.0
FT3 (3.1–6.8 pmol/L)	1.0	3.9	4.3
FT4 (12–22 pmol/L)	1.7	16.3	23.0
TT3 (1.3–3.1 nmol/L)	0.3	1.6	-
TT4 (66–181 nmol/L)	18.6	114	-
TSH (0.27–4.20 μIU/mL)	176.0	21.7	2.2
TPOAb (<34 KIU/L)	>600.0	-	-
TgAb (0–115 KIU/L)	>4300.0	-	-

CHOL = cholesterol, eGFR = estimated glomerular filtration rate, FT3 = free serum triiodothyronine, FT4 = free serum tetraiodothyronine, HDL-c = high density lipoprotein cholesterol, LDL-c = low density lipoprotein cholesterol, TgAb = anti-thyroglobulin antibodies, TPOAb = antithyroid peroxidase antibodies, TRIG = triglyceride, TSH = thyroid stimulating hormone, TT3 = total serum triiodothyronine, TT4 = total serum tetraiodothyronine.

## 3. Discussion

Patients with hypothyroidism often showed abnormal lipid profile such as hypercholesterolemia and hypertriglyceridemia, which was caused by decrease in the number and activity of LDL receptors and lipoprotein lipase activity on the surface of cells.^[5,6]^ Kotwal et al^[7]^ found that hypothyroidism patients with L-T4 therapy exhibited a significant decrease in the serum level of TC, TG, LDL-c, and HDL. Jung et al^[8]^ reported that TC, TG, LDL-c, and HDL levels were significantly increased in the patients with overt hypothyroid and recovered to baseline values with L-T4 replacement therapy. Consistent with previous research, increased TC, TG, LDL-c, and HDL levels of this hypothyroidism patient gradually recovered towards normal value during the L-T4 therapy period. L-T4 replacement can reverse aberrant lipid profile partially because of up-regulation of cholesterylester transfer protein level and increase in low lipoprotein lipase activity.^[5,9]^

Hypothyroidism is one of the rare causes of renal insufficiency. An elevation in the Scr level is usually associated with a reduction in the eGFR. In a prospective study included 30 hypothyroid patients, Scr level decreased and eGFR level increased significantly after replacement with thyroxine for 12 weeks. More importantly, this research found that there is a significant correlation between the change of GFR and the changes of FT4 but not TSH.^[10]^ A retrospective study found that the elevation in FT3, FT4, and eGFR levels and decrease in creatinine and TSH levels were observed after euthyroidism was achieved with levothyroxine treatment. Meanwhile, above-normal Scr levels of some hypothyroidism patients can completely return to normal levels when these patients became euthyroid. However, this study demonstrated that the change of eGFR and the change of TSH levels were negatively correlated before and after L-T4 treatment.^[11]^ Our study found that there was an obvious trend in increase in eGFR level and decrease in Scr level accompanying down-regulation of TSH level with L-T4 therapy in this hypothyroidism patient. Hypothyroidism can result in decreased eGRF mainly caused by impaired renin–angiotensin–aldosterone system activity in association with the following mechanisms^[12]^:

- decreased sensitivity to β-adrenergic stimulus and decreased renin release.
- tubuloglomerular feedback mediated by reduced the renal basolateral chloride channel expression and chloride reabsorption.
- reduced glomerular surface for filtration owing to renal parenchymal growth retardation.

Hence, the renin–angiotensin system may be a factor explaining renal function recovery from L-T4 replacement therapy because thyroid hormones can stimulate renin mRNA in a way that depends on the renin hormone regulatory element.^[13]^

Patients with primary hypothyroidism usually occur muscle injury and about 79% of patients have complaints of muscle weakness, muscle stiffness, spasm, and myalgia,^[14]^ accompanied by elevated serum CK level.^[15,16]^ The elevation of serum CK levels is not completely consistent with the severity of clinical manifestations of myopathy in hypothyroidism patients. However, the elevation of serum CK level may be related to serum TSH level, and the higher the level of TSH, the higher the CK level.^[16]^ Elevation in CK level led by hypothyroidism may be caused by following mechanisms^[17]^:

- Reduction of CK clearance because of decreased T3 level.
- Less ATP generation and increased serum CK that escape from the cell.
- Reduction of the total activity of CK and a compensatory increase in CK level.
- Swelling and breakage of muscle fibers due to the increase of muscle cell volume, the decrease of muscle contraction and a large amount of mucopolysaccharide deposition in the interstitial tissue.

Rhabdomyolysis is one of the most severe complications of hypothyroidism and it can contribute to acute kidney injury with high mortality rates in hypothyroidism patients.^[18]^ The CK level in plasma is related to the severity of rhabdomyolysis, and the risk of AKI development increased when the CK concentrations >5000 U/L.^[19]^ Boryushkina et al^[20]^ reported a rare case that severe hypothyroidism resulted in recurrent uncomplicated rhabdomyolysis. In our study, this patient complained of myodynia and muscle weakness with elevated levels of CK, while rhabdomyolysis did not occur. Moreover, the level of CK recover to normal value and symptom such as myodynia and fatigue were completely were diminished after 3 months of L-T4 treatment.

A recent study found that FT3 <1.3 pg/mL, age at diagnosis of hypothyroidism >60 years and CK >1000 U/L were independent risk predictors for the development of AKI in patients with hypothyroidism.^[21]^ In addition, rhabdomyolysis in hypothyroidism patients correlated with the concomitant statins administration, vigorous exercise, and exposure to excessively high temperatures.^[22–24]^ Future research should place greater emphasis on identifying the risk factors associated with serious complications of hypothyroidism, developing clinical prediction models, and early identification of high-risk population. This will enable more patients to benefit from timely intervention.

Our study had some limitations. Firstly, our research only provided observations from an individual case rather than systematic evidence, which may lead to challenges in generalizing findings to wider populations or predicting outcomes reliably. Then we need further research to explore the risk factors for early detection of patients who are more prone to serious complications. Finally, this patient still needs long-term follow-up to assess the efficacy of treatment.

## 4. Conclusion

Our patient with HT had high levels of Scr, CK, CHOL, TRIG, LDL-c, HDL-c, and low level of eGFR, among which normalized with L-T4 replacement treatment. Kidney injury, muscle injury, and lipid metabolism disorders may occur in patients with HT through a variety of underlying mechanisms and exhibit nonspecific manifestations. Hence, early recognition and proper treatment of the above complications caused by HT are urgent for patients.

## Author contributions

**Conceptualization:** Chenxiang Cao.

**Data curation:** Xuesong Li.

**Funding acquisition:** Chenxiang Cao.

**Methodology:** Xuesong Li.

**Project administration:** Xuesong Li.

**Resources:** Xuesong Li.

**Visualization:** Xuesong Li.

**Writing – original draft:** Xuesong Li.

**Writing – review & editing:** Xuesong Li.
